# Implications of Developmental Levels and Packaging Materials on the Quality of Iceberg Lettuce for Marketing System: A Review

**DOI:** 10.1155/ijfo/5548305

**Published:** 2025-03-17

**Authors:** Tolcha Techane Alemu, Vu Thi Kim Oanh

**Affiliations:** ^1^Jimma University College of Agriculture and Veterinary Medicine, Jimma, Ethiopia; ^2^Vietnam National University of Agriculture, Hanoi, Vietnam

**Keywords:** iceberg lettuce, maturity stages, packaging materials, quality

## Abstract

Iceberg lettuce is one of the most nutritionally important vegetables and plays a great role in economic relevance in the world, being highly consumed both at home and in fast food chains. Additionally, it is valued for its crisp texture and mild flavor. However, the natural perishability of lettuce is facing greater problems during storage and transportation to long-distance marketing. Previously conducted work revealed that the quality of iceberg lettuce is affected by various factors, including maturity stages and packaging materials. This review explores the literature available on the implications of developmental levels and packaging materials on the quality of iceberg lettuce for export-oriented marketing systems. The study provides insight into producers of vegetables and fruits, especially lettuce; harvesting iceberg lettuce at an immature stage could easily damage and deteriorate quickly due to moisture loss and high physiological processes. Overmatured similarly reduces quality because of russet spots, pinking formation, and physiological disorders. Improper packaging materials also affect the quality and shelf life of lettuce through excess loss of water and by restricting the circulation of air, then accumulating high carbon dioxide. Most of the researchers reported harvesting iceberg lettuce on the 59–65 days based on the day after planting and packing with modified atmospheric packaging; corrugated fiberboard with high- and low-density polyethylene liners and plastic crates is very paramount. This review concluded that harvesting iceberg lettuce at the optimal maturity stage and using appropriate packaging materials are very paramount to maintaining the quality of lettuce for marketing, particularly for export purposes.

## 1. Introduction

Iceberg lettuce (*Lactuca sativa* var. capitata) is an Asteraceae family consumed as a fresh perishable vegetable on a global scale [1]. Lettuce is a short shelf life vegetable due to the presence of high moisture, physiological reactions, and easy susceptibility to damage after harvest. Recognized for its nutritional and phytochemical properties and high economic value, iceberg lettuce holds a prominent place among vegetables [2]. Epidemiological studies revealed an association between lettuce consumption and managing diseases because the bioactive compounds (vitamin C, chlorophylls, carotenoids, and polyphenols) are great [2]. This vegetable is native to Asia and is produced widely throughout Europe, the United States, Mexico, Chile, Argentina, Brazil, Peru, China, Japan, and Australia [3]. In South Asian countries, lettuce production has increased, and 17,228 metric tons of lettuce and chicory are produced worldwide, with the primary production of lettuce from China (53%) and the United States (17%) [4, 5]. When vegetables are harvested too early or too late in their development, their quality and yield are decreased due to shrinkage and mechanical damage. The maturation stage of vegetables is the main factor that affects total traits and desirable attributes for export [6]. Packaging is also an additional issue and a necessary element in guaranteeing the standard of management and delivery of vegetables from the grower to the customer [7].

In South Asian and African countries, a variety of vegetables are grown, which are significant components of agricultural economies and boost competitive advantages in international markets during export. However, postharvest handling causes a 20%–44% loss of vegetables due to inappropriate harvesting time and procedures, physiological disorders, postharvest losses (PHLs), packaging, and transportation, and producers are exporting a few in amounts [8]. Nguyen [9] reported that in Vietnam, the PHL of vegetables is 25%–30% due to a weakness in following the harvest management system. Eriksson et al. [10] reported that vegetables are the most lost produce at retail stores, and iceberg lettuce is the third most lost. Demissew et al. [11] also reported that in Ethiopia, PHLs of fruits and vegetables account for 30%–40%. Iceberg lettuce loss (20%–30%) is due to mechanical stress [12]. This crop is difficult to grow, and it suffers problems during exportation due to it being affected simply by climate conditions and physiological processes; as a result, it is not traded much internationally [13]. Transportation time and market distance are the most crucial factors to be considered when making a harvest decision [14]. Currently, the export of iceberg lettuce to international and neighboring markets by air freight has decreased due to increased competition and the high cost of air freight. However, transportation by sea freight is half that of air freight, and this may take time and, as a result, affect the quality of products [15, 16].

Lettuce's sensory quality, color, and phenolic content are influenced by maturity stages [17]. The physiochemical and sensory quality of vegetables was affected during export by sea freight [16, 18, 19]. After harvesting, packaging materials also affect lettuce's sensory quality, physiological parameters, bioactive compounds, and physicochemical qualities, particularly water loss and color change, as well as freshness, which are the main characteristics of external quality that purchasers consider when choosing what they want to buy [20, 21].

The shelf life of iceberg lettuce is shortened by improper packaging, but plastic packaging materials are the most common for vegetable packaging because of their affordability, biochemical resistivity, good moisture barrier, and internal atmospheric modification. Hurley et al. [22] reported that primary and secondary packaging maintains the freshness, quality, and shelf life of vegetables during exportation.

Vegetables are readily damaged and impacted by severe conditions; therefore, special precautions must be taken while transporting them across long distances. The optimal maturity stage and packaging have been considered efficient methods to maintain the quality of iceberg lettuce by decreasing physiological processes and transpiration and determining the quality during cold storage [20, 23]. In particular, proper packaging materials are very important to preserve the quality characteristics of fruits and vegetables during exportation [24]. Hence, optimal harvesting time and appropriate packaging materials play a key role in obtaining iceberg lettuce with better quality and shelf life. However, information regarding the effect of development levels and packaging on the postharvest quality of iceberg lettuce for marketing system export is scarce. Therefore, the goal of this review study is to compile research conducted by numerous researchers on the implications of development levels and packaging materials on the quality of iceberg lettuce for marketing export purposes.

## 2. Literature Review

### 2.1. Developmental Level and Postharvest Characteristics of Iceberg Lettuce

According to Kılıç [25], iceberg lettuce grows rapidly in cooler conditions and reaches optimal maturity in approximately 60 days. However, Soltani Firouz et al. [26] reported that lettuce (*Lactuca sativa* var. longifolia) should be harvested at optimal maturity on the 65th day based on the day after planting (DAP). In another way, B. Chutichudet et al. [17] reported that lettuce var. Grand Rapids should be harvested 59 DAP to reduce incidences of browning and maintain quality. The maturity of a lettuce head is determined by its size and degree of compactness. Similarly, Alemu and Oanh [27] reported that iceberg lettuce of the Saula variety harvested at 58 DAP showed the best quality, and its shelf life lasted for 1 month through the application of appropriate packaging materials.

**Figure 1:**
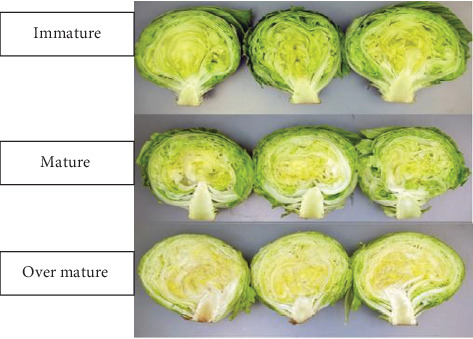
Maturity stages of iceberg lettuce with desirable quality attributes. *Source:* Gil et al. [28].

Researchers reported that the different maturity stages might be due to varieties and soil properties. Gil et al. [28] reported that compared with mature and immature iceberg lettuce, overly mature iceberg lettuce has a worse taste and more postharvest problems. According to research conducted by previous researchers, also, immature lettuce has a very loose head, whereas the overhead of an overmatured one is very firm or hard. Immature heads are more susceptible to injury and have higher respiration rates when fresh [29]. The mature heads have a maximal storage life, whereas the overly mature heads may suffer mechanical damage and acquire pink ribs, russet signs, and physiological disorders. The other main characteristic of the quality of the lettuce is the presence of high moisture, which is active in physiological processes. Different mature stages of iceberg lettuce based on the DAP are shown in Figure 1.

PHL of fresh produce is a quantifiable drop in production in a postharvest system that might be qualitative, quantitative, or economically significant. Singh Meena et al. [30] stated that the majority of quality degradation of vegetables like iceberg lettuce happens after harvest, particularly when marketing by transportation and when there are not enough suitable packaging materials. According to Patil et al. [12], fresh-cut iceberg lettuce is highly perishable with a water content of 95% and results in a short shelf life due to metabolic activities at room temperature. The mechanical stress that occurs during the processing of iceberg lettuce causes environmental factors and cellular delocalization of enzymes and their substrates, which in turn causes biochemical deteriorations such as enzymatic browning, off-flavoring, and texture browning. This is the cause of the PHL of iceberg lettuce.

#### 2.1.1. Causes and Extents of PHL of Iceberg Lettuce

In most developing countries, including Vietnam, PHL is still high (25%–30%) because of the postharvest management system, which makes it difficult to compete for domestic consumption and export markets [9]. PHL of vegetables alone is about 40% [31]. Debebe [32] also reported that PHLs of crops are first for fruit and vegetables (33.38%). There are many causes for PHLs in perishable crops, and they are classified into two main groups: primary causes (physiological factors, biological factors, and physical factors) and secondary causes of losses, which encourage primary factors [33]. Most of the time, the transport and storage of vegetables without packaging for markets result in deterioration [34]. Harvesting factors that cause the loss of iceberg lettuce are as follows:

A. Harvesting stage

The stage of maturity at which iceberg lettuce is harvested significantly impacts its quality, including texture, flavor, and nutritional value. After being harvested, lettuce is susceptible to water loss, wilting, and browning due to its respiration and transpiration. At the same time, its brittleness and high water content make it susceptible to damage during harvesting, transportation, and storage, further hastening its deterioration of quality [35]. Some of the researchers reported that, when iceberg lettuce is harvested at a mature level, it tends to have better flavor and crunch, while immaturely harvested lettuce may be more susceptible to spoilage. Mature lettuce typically has higher water and nutrient density, which can enhance its appeal to purchasers and consumers. The most important stage in determining the postharvest characteristics of fresh produce is harvesting at an optimal mature stage. Harvesting fresh produce without optimal maturity stages and harvesting at the wrong time causes spoilage and loss of quality during the supply chain [36]. Picking vegetables like lettuce at the optimal maturity stage is usually vital to allow for good ripening, transportation over long distances, and long storage life.

B. Harvesting time

The majority of previous research suggested that harvesting timing may have an impact on the quality of vegetables since optimal harvesting times preserve the highest water potential, which causes plants to wilt more slowly than during lower water potential times. Vegetables and fruits should be harvested at optimal maturity stages [28, 37]. Iceberg lettuce is very sensitive to high temperatures. To minimize wilting and the accumulation of fields of heat, lettuce should be harvested during the coldest part of the day, preferably early in the morning or late in the afternoon. Because crisp, brittle leaves shatter easily when handled, lettuce should not be gathered extremely early in the morning when damp or covered with dew. Decay is also more likely in environments with high moisture content. Harvested lettuce should be kept in the shade if packing is delayed.

C. Harvesting methods

Kasso and Bekele [23] stated that the methods of harvesting are the factors responsible for the loss of vegetables. Poor harvesting methods (harvesting by shaking, dropping, twisting, and pulling) at the improper stage and poor care at harvest are some of the reasons that hasten PHLs of perishable crops after harvest. A lettuce vegetable must be cut at the base using a harvesting knife [38]. Old and senescent (yellow) leaves must be trimmed, following which the harvested lettuce must be placed in the field containers.

##### 2.1.1.1. Mechanical Damage

Leafy vegetables are rich in carbohydrates, vitamins, minerals, and other nutrients and are popular in people's daily lives. However, the water loss of leafy vegetables is rapid after harvest due to their large leaves, high water content, and brittle tissues. This makes them susceptible to mechanical damage [39]. According to Mahanti et al. [40], mechanical damage to fresh produce can result in economic loss and is caused by vibrational stress, which can alter the physiological and morphological characteristics of fresh fruits and vegetables. Some of those mechanical injuries may have been caused by improper harvesting techniques, container use, improper trucks, careless loading and unloading, early harvesting, and handling of harvested materials after harvest. Visual defects in fresh produce caused by mechanical damage sustained throughout the farm-to-retail transportation process may significantly reduce the product's overall appearance and lower grower and retailer profits [41].

Lettuce harvested too young or too early is more prone to deterioration and mechanical injury and can have unfavorable characteristics such as low sugar content and high acidity. To reduce the problem of mechanical damage during harvesting iceberg lettuce, knowing the optimal mature stage is crucial. It should be mentioned that, in order to reduce mechanical damage, it is critical to prevent unnecessary bruising, crushing, wounding, or damaging of produce by machinery, containerizing, and packaging materials. Therefore, appropriate postharvest handling practices are essential to avoid different mechanical damage.

##### 2.1.1.2. Water Loss

A significant amount of water is lost from the crops during the physical process of transpiration, which is the primary cause of crop deterioration. Hence, the primary factor contributing to vegetable deterioration is water loss, which causes direct loss of salable weight as well as visual (shriveling and wilting) and textural quality (softening and crispness) losses. Agüero et al. [42] stated that lettuce has a high surface area to volume ratio, meaning moisture is lost when vegetables are harvested at an immature stage and stored below optimal humidity ranges of 95%–98% relative humidity. Particularly, immature vegetables lose a lot of water because of their high surface-to-volume ratios, and the resulting quality is minimized.

Water loss not only induces the important quality, marketability, and economic losses of fresh produce but also negatively influences the visual, compositional, and eating quality of the plant, even when weight losses are subtle. However, water loss from iceberg lettuce could be minimized by maintaining an optimum high atmospheric relative humidity, a low temperature, reduced air movement, avoiding mechanical injury, and using suitable packaging during storage and transportation [43].

##### 2.1.1.3. Microbial Growth

According to Barth et al. [44], the inside tissues of many fruits and vegetables, especially vegetables, are nutrient-rich and have pH values that are almost neutral, which makes them practically ideal for the survival and proliferation of a wide variety of microorganisms (bacteria and fungi). Fruits and vegetables that have been damaged by microbes lose some of their visually appealing qualities, making them available as lower quality products or degraded. The growth of microorganisms such as bacteria and fungi will be at the expense of the vegetables, resulting in weight loss and also damaging the food crop if it becomes unacceptable because of rotting and other defects.

Microorganisms, enzyme interactions, and improper handling cause iceberg lettuce to deteriorate quickly after harvest [45]. Islam et al. [46] claimed that the development of lactic acid bacteria was stimulated by high CO_2_ and low O_2_ concentrations. These bacteria then went on to produce lactic acid, acetic acid, and ethanol as a result of their lactic heterofermentative metabolism, which is how the off odor arose. Microorganisms that affect the quality of iceberg lettuce preharvest and postharvest can be controlled using sanitizer, removing or trimming the outer parts, and using appropriate packaging materials. Enzymatic discoloration is affected by multiple cellular activities, including phenol metabolism, redox reactions, amino acid metabolism, and cell decomposition. The content of total phenolics significantly increases due to the wound-induced accumulation of phenylalanine ammonia-lyase (PAL); however, the higher content of phenolics does not necessarily correlate with the intensity of discoloration [47].

##### 2.1.1.4. Physiological Factors

A. Respiration

Respiration is a physiological process by which fruits' and vegetables' sugars react with oxygen and convert it into carbon dioxide, water, and energy in the form of heat. High temperatures can enhance the rate of respiration and carbon dioxide production in the harvested produce. A lot of researchers reported the temperature of a product is affected by the respiration rate. Kongwong et al. [48] reported that storage temperature should be optimal as the rate of respiration reaction doubles for each 10°C increase in temperature. Fresh-cut lettuce from immature heads produced higher levels of carbon dioxide, which were correlated with off-odor and the accumulation of ethanol and acetaldehyde [49]. However, by reducing the temperature and utilizing the proper packaging materials, the respiration rate can be decreased. One of the factors affecting the respiration rate is the temperature at which the plant is stored. The higher the temperature, the higher the rate of respiration [50]. Therefore, by reducing a plant's respiration rate, its shelf life can be extended.

B. Ethylene

As a plant hormone, ethylene controls several plant processes, including growth, ripening, senescence, and abscission. Furthermore, the synthesis of certain chemicals by horticultural products (such as ethylene) and their buildup in the ambient air speed up senescence processes, hence shortening the product's shelf life. Martínez-Hernández and López-Gómez [51] reported that ethylene gas in plants has effects such as destruction of green color, accelerated senescence and maturation, induction of physiological disorders (e.g., foliar disorders in leafy vegetables), sprouting of tubercles, and abscission of leaves and flowers. It also stimulates the germination of dormant seeds, changes the direction of seedling growth, and can stimulate flowering. The production of ethylene in vegetables and fruits is closely linked to their respiration rate. Measures to reduce the ethylene affinity quality of products include eliminating the sources of ethylene, ventilation, ethylene absorbers such as potassium permanganate, and inhibition of ethylene action such as 1-methylcyclopropene (1-MCP) [52].

##### 2.1.1.5. Loss of Chlorophyll

**Figure 2:**
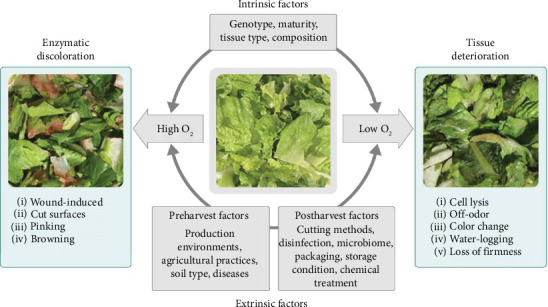
Factors contributing to enzymatic discoloration and rapid tissue deterioration of fresh-cut lettuce. *Source:* Peng and Simko [55].

Chlorophyll exists in many green vegetables, especially in different lettuce varieties. According to Brar et al. [53], increased respiration also results in the loss of chlorophyll in green vegetables. Iceberg lettuce has a high surface area-to-volume ratio and results in the removal of water and color change due to the chlorophyllase enzyme. During storage, the amount of chlorophyll serves as an accurate indicator of leaf senescence. Packaging with appropriate materials and storage under cold conditions are essential for maintaining the chlorophyll content of Iceberg lettuce [54]. Factors affecting the PHL of lettuce are also indicated in Figure 2.

#### 2.1.2. Reduce PHLs of Iceberg Lettuce

Good postharvest handling practices and methods are vital in keeping the quality of vegetables such as lettuce and in ensuring their safety as they move along the marketing chain. Therefore, proper postharvest handling techniques need to be followed in order to guarantee the quality and safety of lettuce for consumers. A lot of researchers reviewed the methods of lettuce fresh-keeping, including physical methods (vacuum precooling, packaging film and air conditioning, low temperature, heat treatment, light, irradiation, and high pressure), chemical methods (disinfectant cleaning and chemical preservatives), and biological methods (biocides and natural extracts) [56]. The preservation of leafy vegetables commonly depends on the application of these technologies [57]. Additionally, other researchers investigated the impact of different packaging materials on the physiology of lettuce and its fresh-keeping quality in the postharvest duration [58].

##### 2.1.2.1. Appropriate Cultural Practices and Variety Selection

The impact of cultural and agricultural practices, such as planting material selection and handling and harvesting techniques on the quality of harvested produce, can lower PHL [59]. Care should be taken to ensure that preharvest procedures like spacing, weeding, and fertilizer applications are done appropriately. Choosing cultivars that are resistant to disease damage, have high nutritional value, and can be kept for an extended period of time without deteriorating after harvest are other ways to reduce PHLs.

##### 2.1.2.2. Proper Harvesting

Proper harvesting maturity refers to the time when the produce (vegetables and fruits) is ready for harvesting. The main reasons for PHLs in most African and Asian countries are at the farm level due to inappropriate cultivar selection and harvesting methods [60]. The maturity stages of harvest are one of the most influential qualities, and the rate of quality changes during postharvest handling and shelf life [61]. Iceberg lettuce should be harvested at the appropriate stage during the cool part of the day, that is, early morning with no wetness and late evening. To ensure a proper harvest, it is important to set an optimal maturity date for the fruits and vegetables. Previously conducted research showed that, because of some perishable vegetables, such as iceberg lettuce, being alive after harvest, they continue to respire, which results in a loss of nutritional value and physicochemical properties. Precooling before storage or distribution can slow down these processes, but they cannot be prevented [62]. Lettuce is lost during the harvesting season due to inappropriate application of cultural practices, inaccurate prediction of harvesting time, harvesting at the wrong time, improper application of harvesting practices, and failure to apply precooling to vegetables.

##### 2.1.2.3. Transportation Practices

Natural perishability, distance, and product value all influence the mode of transportation for fresh produce. In the marketing channel, transportation is frequently the most significant factor. Temperature variations during storage and transportation are the primary determinants of the shelf life of different vegetables. Iceberg lettuce should be polished and placed in primary packaging by the producer as part of the supply chain for exportation. If the lettuce cannot be transported immediately, it is recommended to place it in cold storage temperature to maintain [63]. The chain that connects fresh produce from the field to the customer is facilitated by transportation in multiple ways.

##### 2.1.2.4. Packaging

Packaging ensures that the product is delivered to the end user safely, in good shape, and at a reasonable cost. Keeping and preserving the total traits of products at the time of transfer from one place to another requires proper packaging. The PHLs of vegetables are decreased by packaging, which prolongs shelf life by regulating environmental factors such as temperature and respiration. Fresh produce packaging and storage help to achieve high-quality and safe produce while lowering PHLs at various points in the supply chain from farm to consumer. According to Sualeh et al. [64], improved packaging technologies have a great role in reducing the PHL of vegetables.

##### 2.1.2.5. Proper Storage

The preservation of agricultural products and the prevention of their deterioration for specific durations is an essential aspect of storage, and this practice ensures that the quality of perishable products is maintained beyond their typical shelf life. According to the International Refrigeration Institute, developing countries experience a significant loss of 23% in perishable food items due to the absence of proper cooling facilities [65]. Temperature plays a crucial role in determining the quality attributes of produce such as iceberg lettuce because it directly impacts the rate of biological processes [66]. The previously conducted research revealed the optimal storage temperature and relative humidity for iceberg lettuce storage recommended (0°C–5°C and beyond 95%, respectively). Under this condition, its shelf life is between 21 and 28 days [67]. Additionally, Tolcha and Oanh [68] reported that iceberg lettuce harvested at 58 DAP and stored at 3°C ± 2°C and 95% relative humidity was maintained and extended for 1 month with packaging in a carton box glued with low-density polyethylene (LDPE). According to Mattos et al. [69], storage of iceberg lettuce at the proper temperature and relative humidity could preserve the quality and extent of shelf life by reducing respiration rate, ethylene production, and growth of microorganisms.

#### 2.1.3. Postharvest Handling Operations

Esguerra et al. [70] reported that postharvest operations are the preparation of lettuce for marketing purposes and are listed below with their functions to reduce the PHL of lettuce and maintain quality. Postharvest activities might be conducted in a packinghouse, at collecting centers, or in the field. Postharvest activities need to be performed in a spot that is consistently kept clean and sufficiently shielded from the sun and rain. The packaging area must be kept free of animals. Every employee should practice good personal hygiene and, when necessary, wear appropriate protective gear, including a head covering. These postharvest handling operations are very important before packaging lettuce and should take place carefully and properly.

A. Sorting or grading

Sorting is an essential step in selecting high-quality lettuce. Fresh, somewhat delicate, well-trimmed leaves are a sign of high-quality lettuce; they should also be free of insects, foreign objects, rot, discoloration, and damage. Sorters would maintain proper sanitation by cleaning their hands before starting any sorting. To avoid cross-contamination, the sorting table must be thoroughly cleansed and disinfected both before and after usage.

B. Trimming

**Figure 3:**
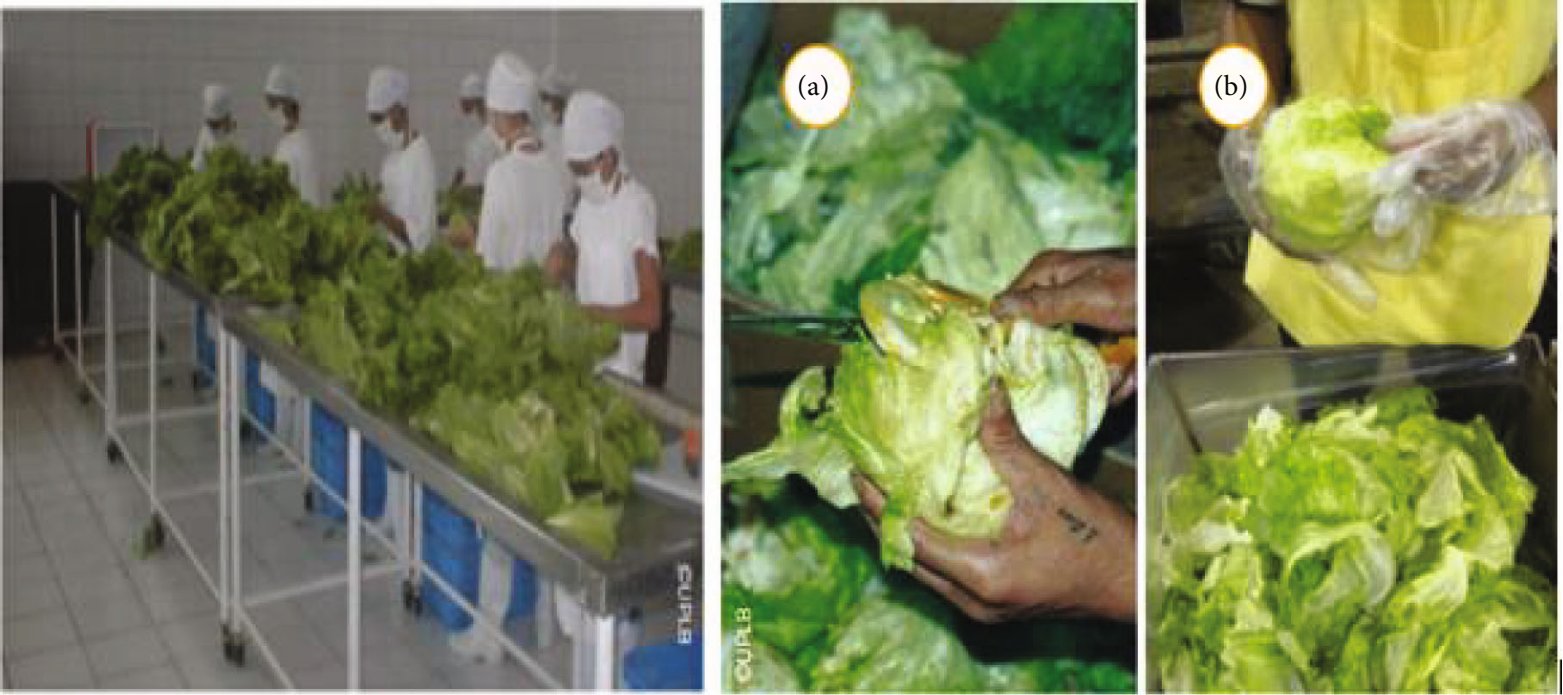
Drying and trimming lettuce destined for high-end markets: (a) trimming lettuce using a knife and (b) gloves being worn during trimming. *Source:* Esguerra et al. [70].

Trimming is the process of removing sections of lettuce that are not desired or that customers are unlikely to accept. Among them are unwanted wrapping leaves. To make the crop more marketable, leaves that are beginning to turn yellow must be removed. Trimming requires the use of a clean knife. Similarly, employees must maintain proper hygiene by washing their hands before cutting. During trimming, it is best to use gloves to reduce the risk of the lettuce becoming infected, as shown in Figure 3.

C. Packaging

Proper packaging materials and methods are very important to preserve the quality of produce during transportation. The primary purpose of packaging is to enable the handling, distribution, and transportation of goods in convenient amounts while offering sufficient protection [71].

#### 2.1.4. Good Practice Using Plastic Crates for Packaging Iceberg Lettuce

Esguerra et al. [70] reported that the following are the key points of good practices that should be followed when plastic crates are used for packaging lettuce. This good practice of using plastic crates for packaging iceberg lettuce is listed in Table 1.

When using packaging materials like plastic baskets, the vehicle should have horizontal dividers built to prevent compression damage [72]. It is critical to control the temperature, particularly in hot, dry conditions. When transporting lettuce in crates, wrap them with polyethylene to stop water loss, which causes withering and loss of marketable weight [73]. The transport vehicle needs to be kept in a hygienic and clean state. When there are decomposing remains of a product from a prior shipment, produce safety is jeopardized [74].

#### 2.1.5. Effect of Maturity Stage on Quality Characteristics of Iceberg Lettuce

Harvest maturity is crucial for quality management and influences the quality of produce. It may also affect how fruits and vegetables respond metabolically. Currently, experts in postharvest practices advise and suggest harvesting iceberg lettuce at its optimal maturity stage to avoid mechanical damage for long-distance marketing and to reduce quality problems. The study by Nadeem and Zeb [75] showed that the physiological characteristics and phenolic compound quality of vegetables are influenced by the mature stage. It also affects vegetable sensory qualities and shelf life. In contrast, for vegetables harvested at the appropriate maturity stage, those harvested too early or too late cause postharvest physiological issues, as well as loss of product and nutritional value [76]. Immature heads of iceberg lettuce are more susceptible to injury and have a high respiration rate in the fresh market. Overly mature heads are more susceptible to mechanical damage, russet spotting, pink ribs, and other physiological diseases. Mature heads have the longest storage life. According to research by Kang et al. [29], overmature lettuce had a higher browning rate than immature and mature lettuce; compared to mature and overmature lettuce, the rate of degradation was greater in immature lettuce.

### 2.2. Packaging Materials and Technology

It is crucial to preserve the quality of iceberg lettuce from the time it reaches the production facility until it is consumed by the final customer. There are many packaging materials and methods for the preservation of agricultural products, such as vegetables including lettuce. Some of the packaging materials used for packaging vegetables, including lettuce, are very thick, and others are very thin; as a result, they affect quality and shelf life. If packaging materials are thick, there is no regulation of gases inside to outside, and this might lead to the oxidation process and accumulation of higher CO_2_. However, if packaging materials are very thin, the moisture could easily be removed, and quality is reduced. Although a common packaging material for packing different fruits and vegetables, including lettuce, such as polyethylene, cardboard boxes, and plastic boxes, could extend the shelf life of fruits and vegetables [77]. Particularly, plastic packaging materials are often used for their moisture retention, thus used in export-related transportation. Polyethylene plastic films are frequently favored for packaging because of their low water permeability characteristics. To maintain the general quality, freshness, appearance, and color of the product, a packaging material with the right permeability, thickness, and strength should be selected [78].

#### 2.2.1. Primary Packaging Materials and Packaging Technology

**Figure 4:**
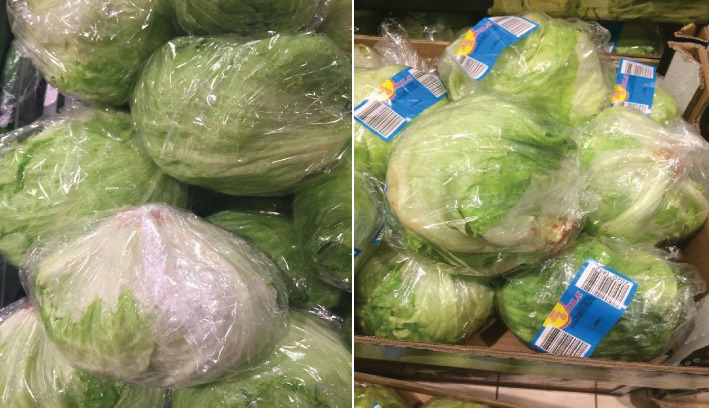
Primary packaging for iceberg lettuce. *Source:* Mansner and Wang [79].

Primary packaging is the level of packaging in which the layer of the packaging is in direct contact with the products usually offered to customers. It is the most important packaging level regarding extending a product's shelf life. Primary packaging is the level of packaging in which the layer of the packaging is in direct contact with the products usually offered to customers. It is the most important packaging level regarding extending a product's shelf life. Primary packaging materials for lettuce are shown in Figure 4.

##### 2.2.1.1. LDPE

Among all the packaging materials, LDPE was found to be the most effective at preventing weight loss and preserving vegetables for better quality over time [80]. Lee and Chandra [20] reported that perforated polypropylene (PP) with 1320 small-sized holes, perforated PP with four large-sized holes, nonperforated PP, and nonperforated PP with antifogging properties stored at 10°C extend the shelf life of lettuce up to 16 days. Furthermore, they have reported that antifog-PP treatment could provide better postharvest qualities along with extended marketable life for about 2 weeks during storage at market display temperature. Thus, when combined with cold storage, plastic packaging can extend the shelf life of vegetables. In particular, cold storage of fresh food in polyethylene can help maintain moisture and freshness [81]. The work by Paulus et al. [82] showed that okra packed in LDPE at 10°C maintained its quality for 28 days. LDPE has strong moisture barrier qualities, is poorly odor-resistant, and is chemically inert. According to Paneru [83], bell peppers packed in LDPE under ambient conditions have a 32-day shelf life. Sualeh et al. [84] showed that the highest weight loss was recorded for tomatoes stored in the ambient atmosphere without packaging (11.68%), whereas the lowest loss was recorded in refrigerators with LDPE packaging (1.67%) after 24 days. Bark et al. [85] demonstrated in a 3-week storage period at 2°C that lettuce wrapped in LDPE film within a plastic box container had the least amount of weight loss and the best appearance when compared to lettuce treated with different packing and storage methods.

##### 2.2.1.2. High-Density Polyethylene (HDPE)

Previously conducted research revealed that, in addition to providing a superior barrier to gases and moisture, HDPE is more brittle, tougher, thicker, and less flexible than LDPE. HDPE sacks are watertight, chemically resistant, and have excellent tear and puncture resistance. It is frequently used in conjunction with other films rather than alone. According to Poudel et al. [86], HDPE was found to be the most effective packing solution for preventing fungal growth, minimizing weight loss, and preserving tomato quality for an extended period of time. According to Sualeh et al. [84], the highest weight loss was recorded for the tomatoes stored in an ambient atmosphere without packaging (11.68%), while the least loss was recorded in cold storage in HDPE packaging (1.67%) during storage for 24 days.

##### 2.2.1.3. Modified Atmospheric Packaging (MAP)

MAP is an appropriate packaging technology that maintains and preserves the quality characteristics of products. Zahirul et al. [87] found that this packaging technology, when combined with a low storage temperature, can minimize PHLs of leafy vegetables by creating a suitable environmental condition within the container that preserves the quality and minimizes spoilage. MAP regulates the gas composition within a package and thus extends the shelf life of harvested fresh produce. Therefore, MAP is the best choice for low-temperature storage regarding keeping the quality of fresh products [88]. MAP could extend the shelf life of Chinese cabbage to 25°C in an atmosphere of 2%–5% O_2_ and 3%–10% CO_2_ for 5 days [35, 65]. For romaine lettuce, the levels of initial O_2_ are recommended to lie between 0.5 and 3.0 kPa [89]. Mattos et al. [90] reported and suggested fresh-cut lettuce should be stored under MAP with gas concentrations of O_2_ (0.5%–3%) and CO_2_ (10%–15%) during storage at 0°C–5°C. The effect of MAP on the shelf life of fruits and vegetables is shown in Table 2.

##### 2.2.1.4. Polyvinylchloride (PVC)

PVC films provide a good gas barrier and moisture and odor control qualities. To increase the barrier qualities of various films and bottles, PVC was also applied as a coating. Chaiprasart [97] concluded that “modified atmosphere packaging using PVC film wrapping might be more effective for extending the shelf life of the lychee fruits,” particularly in maintaining weight. Additionally, Dladla and Workneh [98] also concluded that the “expandable polystyrene + polyvinylchloride wrap at cold storage conditions” preserved the quality attributes of tomatoes by hindering the physiological and biochemical processes and extending their shelf life with the lowest weight loss with a 0.23% value on Day 7. The lettuces wrapped with PVC film had lower weight loss, color change, and browning index and the highest total phenolic content compared with lettuces packaged in corrugated and plastic boxes. According to Chang and Lee [99], wrapping commodities in PVC film for storage lasted for 21 days without compromising freshness.

##### 2.2.1.5. PP

PP has good chemical resistance, low moisture permeability, low moisture absorption, fat-resistant films, and moisture-proof wrapping. Akbudak [100] reported that PP extends the shelf life of products by delaying physiological processes and delaying ripening. According to Ngoc and Thi Thanh Que [101], PP maintained the sensory properties and reduced the weight loss of Ceylon spinach during 7 days' storage at 10°C ± 1°C.

#### 2.2.2. Secondary Package

**Figure 5:**
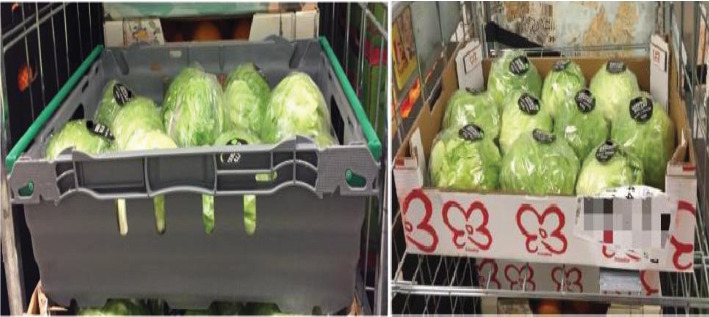
Secondary packaging materials for lettuce. *Source:* [67].

Secondary packaging is a form of multiple packaging that is used to handle packages together. It holds the primary packaging during transport and storage, thus facilitating its handling. Secondary packaging materials have a great role in protecting the stuffing during transport and distribution for marketing [102]. The work by Kim et al. [103] showed that the endive wrapped with HDPE film inside the paper box showed the lowest weight loss and the best appearance compared to those wrapped in other packaging types during storage. Pallets are required to support the weight of the product shipped on them. Secondary packaging materials for lettuce during exportation are shown in Figure 5.

##### 2.2.2.1. Corrugated Fiber Board

Lockrey et al. [81] reported that secondary packaging, such as corrugated fiberboard, is essential for vegetables for protection, promotion, convenience, information, handling, and safety. Corrugated fiberboard with HDPE and LDPE liners, crates, and wooden boxes maintained the physicochemical properties of pears during storage in the cooling chamber at 0°C–1°C and 90%–95% [104]. Because the corrugated fiberboard is often open and cannot be piled on top of it, it is frequently used to hold the iceberg lettuce when it is packaged in the truck. For instance, open corrugated fiberboard would benefit from having a protective layer of material to cover the opening. The hole in packaging materials helps make air circulate. Iceberg lettuce can also be stored in carton boxes because they keep the lettuce warm without overheating it. According to Olveira-Bouzas et al. [105], tomatoes were stored at 6°C and packed in MAP in cardboard box pallets to extend their shelf life, delay color loss, and reduce texture loss. Concerning the cardboard box as the secondary packaging, it is possible to have shelf-ready packaging, which would reduce double handling when replenishing the retail stores and decrease the risk of damage [106]. Also, the secondary packaging contains useful information such as origin, weight, and brand that consumers might be interested in knowing.

##### 2.2.2.2. Plastic Box

Selecting the logistics solutions that best suit the vegetables' respiration and perspiration processes throughout transfer and transportation is crucial to extending the shelf life of fruits and vegetables from the time of harvest until they are delivered to stores. Previous studies revealed that most users thought that using plastic crates to carry vegetables would result in a supply of high-quality vegetables. Vegetables such as iceberg lettuce are well maintained in plastic containers because of their strength and ability to keep moisture out of the container while lowering the risk of mechanical damage. Extra packaging material is required when using returnable plastic containers to prevent iceberg lettuce from making wide holes in the containers [96]. The work of Farahanian et al. [21] showed that plastic carton packaging materials maintained the quality parameters of tomato fruits during storage.  Table 3 shows some packaging materials and technology for lettuce, including their advantages and disadvantages.

### 2.3. Research Status on the Effect of Maturity Stages and Packaging on the Quality and Shelf Life of Iceberg Lettuce

Owing to its naturally high perishability, lettuce may suffer flavor deterioration, yellowing, softening, and shrinking as it travels through the supply chain and is displayed to customers at retail stores [110]. Most scholars have reported that the quality of vegetables is influenced by mature stages, particularly if harvested and picked at an immature level. They are not mature to a satisfactory color and flavor; they are susceptible to shriveling and mechanical damage and develop poor sensory quality, resulting in a loss of consumer confidence. According to Sharma et al. and Mohammed and Brecht [36, 111], the optimum maturity stage for vegetables such as lettuce is essential to maintain quality for marketing purposes. In addition to microbiological degradation, the postharvest quality and shelf life of lettuce are also adversely affected by changes in texture. Another important factor influencing the qualitative characteristics of lettuce is transpiration and respiration. However, packaging would be helpful to give the customer a fresh product; prolong the product's shelf life, which preserves the qualities of lettuce; and restrict the negative changes by lowering transpiration, oxidative stress, and bacterial growth [36].

Manolopoulou et al. [112] also showed that packaging materials, storage time, and temperature affect the quality of romaine lettuce. According to their reports, in order to prevent quality loss, it is highly recommended to pack freshly cut romaine lettuce with MAP at low temperatures. Numerous studies have demonstrated that various forms of polymeric film packaging can effectively impede the processes that lead to the deterioration of fresh vegetables' quality during storage, including the reduction of shriveling, the minimization of color changes and weight loss in leaf lettuce, the reduction of CO_2_ accumulation, the off-odor and decay of fresh-cut salad savoy, and the extension of *Gynura bicolor*'s storage life [113]. However, most polymers reduce the water vapor transmission rate of products. The accumulation of excessive relative humidity inside packaging materials due to lower permeability causes moisture condensation, CO_2_, microbial growth, and product deterioration [114].

#### 2.3.1. Effect of Maturity Stages and Packaging on Sensory Properties

Iceberg lettuce quickly loses its quality when harvested before fully maturing, making it vulnerable to both mechanical damage and physiological disorders. Tudela et al. [49] found a better appearance and less cut-edge browning of heads processed at the immaturity stages versus the matured ones. Immature iceberg lettuce had a high correlation with the production of off-odor due to higher CO_2_ and accumulation of ethanol and acetaldehyde. As a result, it was concluded that the mature stage influences the sensory properties of vegetables. Unlike fruits and vegetables, leaf lettuce loses water and freshness quickly after harvest, which results in the deterioration of the cell wall and other quality characteristics such as texture, color, and other sensory and nutritional qualities [115]. Packaging materials also influence the sensory characteristics of vegetables because lettuce respiration intensity and product color change depending on the composition of the materials used. As a result, different mature stages have an impact on the quality of vegetables. In particular, if vegetables are harvested at an immature stage of maturity, they may shrivel, sustain mechanical damage, and develop poor sensory qualities (color, flavor, and taste). They may also have an impact on color, weight, total soluble solids (TSS) (good flavor), and phenolics, which may cause consumers to lose faith in the product during marketing [17, 37].

According to Gil et al. [28], if iceberg lettuce is harvested at an overly mature stage, it exhibits the worst bitterness, and its color changes due to enzymatic activity. Iceberg lettuce, in particular, experiences a decline in its quality and sensory attributes even before it reaches domestic and international markets via marine transport, leading to substantial market quality deterioration [24]. The work by Chang and Lee [116] showed that the sensory quality was evaluated based on the freshness, color (leaf yellowing), and decay qualities of the samples. The unpacked control sample lost visual quality, and the samples packed with antifog-PP treatment continued to have the highest quality due to the modification of the inside atmosphere of leaf lettuce because lettuce respiration intensity and product color changes depending on the content of the materials utilized. Packaging materials also affect the sensory qualities of products.

#### 2.3.2. Effect of Maturity Stages and Packaging on the Respiration Rate

Leafy vegetables continue to respire even after harvest [117]. Islam et al. [118] reported that smaller fruits and vegetables produce more ethylene and respire at higher rates because they have larger surface area-to-volume ratios than larger fruits and vegetables. Because of its beneficial effect on lowering the rate of respiration and metabolism, lowering the O_2_ level within the package can extend the shelf life of different vegetables and fruits. The respiration rates of the whole and fresh-cut lettuce were 29.5% ± 3.2% and 35.3% ± 6.7%, respectively, showing similar results to those reported for romaine lettuce at 30.38 mL CO_2_/kg/h at 20°C [119]. The process of respiration diminishes the number of nutrients in the plant and has a detrimental effect on flavor, sweetness, weight, and water content.  Table 4 shows respiration rates and ethylene production of lettuce.

#### 2.3.3. Effect of Maturity Stages and Packaging on the Physicochemical Properties

##### 2.3.3.1. Color

The primary characteristic of leafy vegetables that contributes to their visually appealing qualities and is correlated with their chlorophyll concentration is their color [121]. Vegetable color is another crucial quality factor that affects vegetable purchases. Enzymatic browning is the process by which enzymes convert phenolic compounds found in fruits and vegetable tissues to quinone, which are then polymerized to form brown substances and cause discoloration. Reactive oxygen species, phenolic compounds, and phenolic enzymes are the three prerequisites for enzymatic browning. Quamruzzaman et al. [37] observed that despite being harvested at an early stage of maturation, they have poor flavor and are prone to shriveling and mechanical damage. Lee and Chandra [20] found that by regulating enzyme activity and environmental factors, packaging materials preserved the color change of lettuce. The decline in hue in green vegetables such as green or red leaf lettuce is a common phenomenon as the lower hue value indicates an increase in leaf yellowing, which is associated with the degradation of chlorophyll content [122]. The change of color lettuce is presented in Table 5.

##### 2.3.3.2. Weight Loss and Firmness

**Figure 6:**
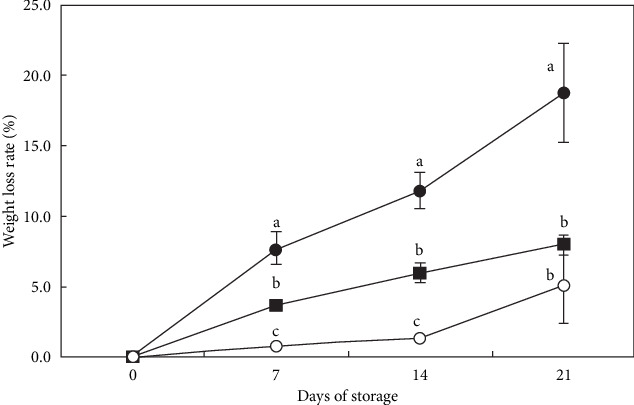
Changes in the weight loss rate of lettuce by different packaging methods during 21 days at 2°C. ■, corrugated box; ●, polypropylene (P) box; ○, individual wrapping using polyvinylchloride (PVC) film. Means that do not share a letter are significantly different (*p* < 0). *Source:* Chang and Lee [128].

Quality characteristics of vegetables, such as fresh-cut iceberg lettuce, are affected by their weight and firmness. Numerous studies have shown that film wrapping can prevent water loss and preserve the quality of perishable crops. The study by Moreira et al. [123] indicated that lettuce leaves stored at a low temperature (0°C) showed no more quality changes, less microbiological infection, or changes in other physicochemical parameters such as weight loss and firmness. Because of its high surface area-to-volume ratio, iceberg lettuce is especially vulnerable to moisture loss, which can cause it to wilt and shrink. The increased weight loss and decreased firmness during storage are mainly caused by respiration and the consumption of organic matter [124]. There is an increase in weight during plant development [18, 125]. According to Agüero et al. [126], wilting, shriveling, and softening are signs of water loss, which is the main indicator of deterioration in lettuce quality. Martínez-Sánchez et al. [127] reported that very loose heads of lettuce are immature and very firm or hard heads are overly mature. The weight loss of lettuce across storage time is shown in Figure 6.

##### 2.3.3.3. TSS

Most of the research conducted has shown that a quality indicator known as TSS is used to assess the marketability, sweetness, ripeness, and maturity stages of fruits and vegetables. Fruits and vegetables that are harvested at the mature stage of maturity have excellent flavor, but they do not keep well and are not suitable for long-distance transportation. Fruits and vegetables that are harvested before they reach their full potential may remain green for a longer period of time, but they might not mature to a suitable color and flavor, which could cause consumers to lose their confidence in them [76]. This study's findings showed that TSS changes as maturity increases, which is explained by the increased respiration that results in sugar metabolism. According to Filho et al. [129], variations in metabolism and respiration rate across maturity stages may be the cause of the fluctuation in fruit soluble solid content. Low TSS was most likely caused by the preservation of a humid microclimate inside these films, which delayed ripening and led to a slow breakdown of proteins, lipids, carbohydrates, and pectic acid, thus low TSS [104].

#### 2.3.4. Effect of Maturity Stages and Packaging on the Bioactive Component

##### 2.3.4.1. Phenolic Compounds and Vitamin C

Phenolic compounds are a class of antioxidants produced by plants to protect themselves against infections and stress. B. Chutichudet et al. [17] reported that in lettuce, phenolic decreased and vitamin C increased with maturity stages. Several studies have also reported that the degradation of phenolic compounds may be affected by storage temperature [130]. The result reported by Liu et al. [131] showed lettuce cultivated in Colorado had a higher TPC than that of iceberg lettuce purchased from local supermarkets, which has a TPC value of 10.4 mg/GAE/gFW, and crosshead-type lettuce drubal variety has 24.8 mg GAE/100 gFW. According to Liu et al. [132], vitamin C levels in fresh-cut lettuce that were vacuum-packed had the highest values (12.5 mg GAE/100 g) at Day 0 and decreased during storage due to oxidation and environmental factors. According to Patil et al. [12], ascorbic acid comprises 55%–65% of the total vitamin C content in freshly cut iceberg lettuce. They also reported that the initial ascorbic acid concentration of lettuce leaves ranged from 11.61 to 11.82 mg/100 g. Vitamin C levels may have decreased because of the water affinity of ascorbic acid, heat degradation, increased surface area, injury, and enzymatic oxidation during storage. The total phenolic content for different varieties of lettuce is presented in Table 6.

##### 2.3.4.2. Chlorophyll and Carotenoids

Leafy vegetables that undergo minimal preparation may lose more of their greenness because of chlorophyll leakage and chlorophyll enzymes. In particular, for leafy green vegetables, maintaining the chlorophyll content is essential for preserving the freshness of the produce. There have also been previous reports of variable patterns of change in chlorophyll concentration during the storage of vegetables and leaves [134]. Chlorophyll a, b, and total chlorophylls (total chl) (6.13, 1.21, and 7.34 *μ*g/mL), respectively, were packaged under MAP for red romaine leaf lettuce during storage at 8°C, and the total carotenoids (41.20 mg 100 g/FW^−1^) were reported in previous reports [135]. Lee and Chandra [20] also reported that packaging materials are important for maintaining pigments and Chla, Chlb, and total concentrations of 0.59, 0.14, and 0.73 mg/g FW for lettuce, respectively. Another quality indicator of fresh lettuce is carotenoids. However, lipoxygenase and other enzymes help to enhance the oxidation of carotenoids, which is induced by exposure to oxygen because of double bonds and lipid-soluble substances.

Previously conducted research revealed the reduction of the concentration of carotenoids in vegetables during processing might be due to loss of moisture. The chlorophyll content of lettuce during storage at 4°C is shown in Table 7.

#### 2.3.5. Effect of Maturity Stages and Packaging on the Decay Rate

Peng et al. [137] found that when fresh-cut lettuce was packed in a modified cool environment, younger leaves deteriorated more slowly than mature leaves. However, oppositely, Costa et al. [138] found that immature iceberg lettuce deteriorated more quickly than more mature iceberg lettuce. According to Kaur et al. [104], the highest deterioration was observed in boxes made of wood (6.91%), followed by crates (6.3%) and corrugated fiberboard boxes (5.8%). These rates were significantly higher than those of the other packaging methods, which may have been caused by elevated CO_2_ and restricted water flow. According to Kang et al. [29], immature lettuce spoiled more quickly than mature and overmature lettuce. The rates of respiration and metabolic activity, which are frequently higher in younger leaves, have been linked to the rate of deterioration.

A benefit of multileaf lettuce compared with baby lettuce is its advanced maturity stages, which confer higher firmness and longer shelf life.

### 2.4. The Supply Chain of Iceberg Lettuce and Consumer Preference

One way of increasing the shelf life of vegetables, according to Verghese et al. [106], is to prepack and process food, which would lead to a reduction of waste in the distribution as well as at the consumer stage of the supply chain. The supply chain actors should work together to get a better understanding of the wastage that occurs in the chain, and by collaborating among actors, new technology, packaging, and information sharing can be used to reduce unnecessary or out-of-date stock. According to Mansner and Wang [67], in production, after harvesting, iceberg lettuce is polished and packed in primary packaging by the producer, and if the lettuce cannot be transported immediately, it will be placed in cold storage, which has a temperature of around 4°C. After that, the lettuce is packed in secondary and tertiary packaging before being transported by truck from the producer to the distribution center or straight to the retailer. After the iceberg lettuce is received at the distribution center, it is placed in cold storage, with a temperature of around 4°C, for a short period of time [139]. The temperature in the trucks delivering products to the retailers varies depending on the requirements of the different retailers. There is different regulatory compliance to export lettuce [67].

**Figure 7:**
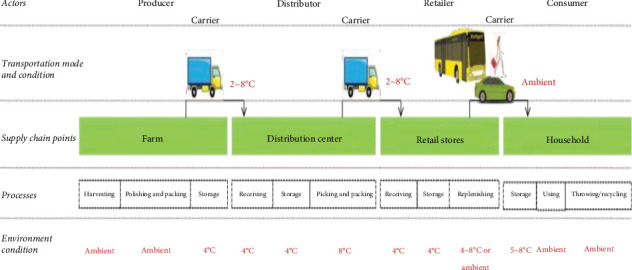
The supply chain of iceberg lettuce. *Source:* [67].

Consumer demand for higher quality and nutrient-dense fresh vegetables is increasing. Therefore, superior varieties and improved cultivation methods are urgently needed to improve vegetable-like lettuce quality. Fresh lettuce is a desirable but highly perishable product, the acceptance of which by consumers may be severely reduced due to external blemishes affecting its overall appearance [140]. The 2022 Chinese Dietary Guidelines recommend a daily intake of > 300 g of fresh vegetables [141]. Overall quality is a key factor in fresh vegetable consumption, which influences consumer purchasing. Previous studies have highlighted the protective roles of vegetable consumption against many chronic diseases, such as ischemic and hemorrhagic strokes, ischemic heart disease, and certain cancers [142]. Therefore, greater effort is needed to improve the appearance, taste, aroma, texture, and nutritional value of lettuce to meet the increasing demand for high quality. The supply chain of iceberg lettuce is indicated in Figure 7.

## 3. Conclusions

This review paper compiled different research works on the implications of developmental levels and packaging materials on the quality of iceberg lettuce for export-oriented marketing systems. The developmental stages and packaging materials influence the quality and shelf life of iceberg lettuce. However, harvesting iceberg lettuce at the optimal maturity stage, 59–65 days based on the DAP; packing with packaging materials (primary and secondary packaging materials), like corrugated fiberboard with HDPE and LDPE liners and plastic crates; and appropriate packaging technology, such as MAP technology, is really essential to maintain the general quality and shelf life, especially for exportation purposes during the commercialization system. This review concluded that the quality and shelf life of iceberg lettuce harvested at optimal developmental stages and packed with effective packaging materials extend the shelf life by protecting lettuce from damage (physical damage) and reducing moisture loss, physiological factors (respiration and ethylene production), and enzyme activity. The good permeability of packaging materials allows for gas exchange that can help maintain visual quality, such as freshness, color, texture, and absence of blemishes, and reduce spoilage, as a result increasing marketability. Marketing implications such as consumer preferences, supply chain considerations, and regulatory compliance should be considered to provide lettuce for marketing systems, such as for exportation. Future research should focus on innovative packaging solutions and optimal harvesting practices to further enhance the quality of iceberg lettuce in the marketing system.

## Figures and Tables

**Table 1:** Some good practices when using plastic crates for packaging lettuce.

**Good practice**	**Consequences**
Hygiene	Before use, plastic crates should be properly cleaned with sanitizer. The microbiological load in the plastic crate is decreased by disinfectants such as sodium hypochlorite.
Handling	Care and attention are important when loading, stacking, and unloading plastic crates.
Storage	Plastic crates need to be kept in a hygienic location to keep rodents and insects out.
Transport	Ensuring lettuce does not get harmed or lose water while being transported is one of the primary goals. Before going to the market, lettuce typically travels through three stages of transportation: (I) from farm to collecting center, (II) from packing shade to whole market, and (III) from whole markets to retail.

**Table 2:** Effect of MAP on the shelf life of fruits and vegetables.

**Fresh produces**	**Shelf life in MAP**	**Shelf life at normal temperature**	**References**
Cherry 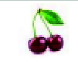	30–40 days	7–14 days	[91, 92]
Fig 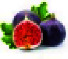	28 days	Less than 14 days	[93, 94]
Grape 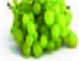	7 days	4 days	[95]
Iceberg lettuce 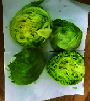	21–28 days at 0°C and 95% relative humidity	4–5	[96]

*Note: Source:* Elik et al. [65].

**Table 3:** Advantages and disadvantages of different packaging materials/technologies on lettuce.

**Packaging materials/technologies**	**Advantage**	**Disadvantage**
Modified atmosphere packaging (MAP)	Preserve quality and extend the shelf life	Requires educated person (specialized knowledge and equipment), expensive, and not eliminate all pathogens
Polyethylene (PE)	Good barrier to moisture and water vapor	They are not a particularly high barrier to gases such as carbon dioxide and oxygen
Low-density polyethylene (LDPE)	Excellent barrier for water vapor	Very bad O_2_− barrier characteristics
High-density polyethylene (HDPE)	Excellent barrier for water vapor. Also, oxygen barrier characteristics better than low-density polyethylene	Opaque
Plastic box	Long shelf life and not breakable	It sweats to produce and affects quality
Wood box	Protect damage of produces.	Absorb moisture, breakable
Corrugated fiber board	Protection like waterproofing, promotion, convenience, information, handling, and safety and not weatherproof	Affected by moisture and can be affected by extreme weather such as rain and snow, deformed or lose its shape if under extreme pressure
Carton box	Protect mechanical damages, reducing environmental impacts, and improved product preservation	It may be torn, not for heavy items, deforms under pressure, and is not weatherproof
Polypropylene	Good appearance and chemical resistance, low moisture permeability, and moisture-proof wrapping	Low UV resistance, nonbiodegradable, difficult to recycle, and low heat resistance

*Note: Source:* [20, 107–109].

**Table 4:** The respiration and ethylene production of lettuce.

**Lettuce**	**Respiration rate (CO**_**2**_ ** mL/kg·h)**	**Ethylene production rate (C**_**2**_ **H**_**4**_ ***μ*L·kg** ^**−1**^ **·h** ^**−1**^ **)**
Whole	29.5 ± 3.2	0.011 ± 0.002
Fresh Cut	35.3 ± 6.7	0.015 ± 0.003

*Note: Source:* Choi et al. [120].

**Table 5:** The interaction effects of nanopackaging and storage time on the color parameters of lettuce in MAP.

**Storage time (day)**	**L**∗** value**	**a**∗** value**	**b**∗** value**	**Color change (**Δ**E)**
1	85.62 ± 2.73	6.97 ± 0.66	23.12 ± 1.98	—
3	84.73 ± 1.21	8.41 ± 1.13	33.33 ± 1.56	2.41 ± 0.43
5	80.43 ± 2.66	8.12 ± 1.25	23.98 ± 3.22	6.59 ± 1.29
9	75.59 ± 0.65	9.32 ± 1.10	27.22 ± 1.46	11.38 ± 1.56
15	59.70 ± 8.29	11.06 ± 1.32	28.87 ± 2.76	27.14 ± 2.19

*Note: Source:* Farahanian et al. [21].

**Table 6:** Total phenolic acid content in different types of lettuce.

**Lettuce type**	**Variety**	**Total phenolic acid contents (mg/100 g FW)**
Loose leaf type	Oak leaf	66.3
Butterhead type	Red salanova	21.7
Crisp head type	Asdrubal	80.4
Romaine type	Cazorla	65.3
Butter head type	Green salanova	5.9

*Note: Source:* Yang et al. [133].

**Table 7:** Average content of chlorophyll (mg g^−1^ fresh weight) during the storage period at 4°C.

**Chlorophyll content/lettuce type**	**Storage (days)**		
	**0 day**	**3 days**	**7 days**
Lettuce—*Lactuca sativa* L. var. capitate	25.80 ± 0.93	18.26 ± 1.19	13.26 ± 1.48
Curly lettuce—*Lactuca sativa* L. var. crispa	29.46 ± 2.71	22.77 ± 1.54	21.40 ± 1.10
Arugula—*Eruca sativa*	0.93 ± 0.74	26.19 ± 0.92	23.10 ± 1.04

*Note: Source:* [136].

## Data Availability

All relevant data for the work are included in the manuscript.
